# Plasma and Synovial Fluid VEGF‐A and Neuropilin‐1 in Knee Osteoarthritis: Associations With Radiographic Severity, Pain Sensitization‐Related Features, and Disease Burden

**DOI:** 10.1002/kjm2.70262

**Published:** 2026-07-15

**Authors:** Cheng‐Ju Tan

**Affiliations:** ^1^ Acupuncture and Tuina Department Affiliated Sports Hospital of Chengdu Sport University Chengdu China

**Keywords:** knee osteoarthritis, Neuropilin‐1, pain sensitization, synovial fluid, VEGF‐A

## Abstract

This study aimed to investigate associations of plasma and synovial fluid vascular endothelial growth factor A (VEGF‐A) and neuropilin‐1 (NRP1) levels with radiographic severity, pain sensitization‐related features, and disease burden in knee osteoarthritis (KOA). Plasma VEGF‐A and NRP1 were measured by sandwich ELISA in 120 patients with KOA (Kellgren–Lawrence [KL] grade 2, *n* = 40; grade 3, *n* = 52; grade 4, *n* = 28) and 53 healthy controls, while synovial fluid VEGF‐A and NRP1 were assessed in the KOA group. Pain sensitization–related features and disease burden were evaluated using the Central Sensitization Inventory (CSI), pressure pain threshold (PPT), Western Ontario and McMaster Universities Osteoarthritis Index (WOMAC), and visual analog scale (VAS). Plasma VEGF‐A and NRP1 levels were elevated in KOA patients versus healthy controls and increased with KL grade, while synovial fluid VEGF‐A and NRP1 rose with radiographic severity. Synovial fluid VEGF‐A showed stronger discriminatory performance for radiographic severity than synovial fluid NRP1. After the Benjamini–Hochberg FDR correction, plasma and synovial fluid VEGF‐A/NRP1 remained associated with VAS, WOMAC, CSI‐defined symptom burden, and local PPT in the overall KOA cohort. Multivariable regression adjusted for KL grade, age, sex, BMI, and symptom duration showed that synovial fluid VEGF‐A had the strongest independent associations with clinical outcomes. Bootstrap‐validated combined ROC models, particularly those incorporating VEGF‐A, showed good discriminatory performance for KOA status and severity stratification. These findings suggest that the VEGF‐A–NRP1 axis, particularly synovial fluid VEGF‐A, may serve as a biomarker framework for KOA severity assessment and symptom‐related phenotyping.

## 1 Introduction

Osteoarthritis (OA) is the most common degenerative joint disorder and a major cause of chronic pain, physical disability, and reduced quality of life in middle‐aged and older adults [1, 2]. Among its various phenotypes, knee osteoarthritis (KOA) is the most prevalent and accounts for more than half of all OA cases worldwide [3]. Previous clinical studies in patients with OA have also shown that pain‐related phenotypes, including neuropathic pain features, are associated with impaired functional capacity and poorer quality of life [2]. As populations age and obesity becomes increasingly common, the global burden of KOA continues to rise. According to the Global Burden of Disease 2021 Osteoarthritis Collaborators, the age‐standardized prevalence of KOA reached 4307.4 cases per 100,000 population in 2020, and the number of affected individuals worldwide is projected to increase to 642 million by 2050, representing a 74.9% increase compared with 2020 [4]. Current diagnosis and severity assessment of KOA rely mainly on clinical manifestations and imaging findings. However, radiographic changes do not always parallel symptom severity, particularly pain and functional limitation. In addition, conventional imaging is limited in its ability to capture early biochemical and microenvironmental changes within the joint. For this reason, there is growing interest in identifying biofluid‐based biomarkers that may reflect pathological activity more directly and objectively, especially in synovial fluid and blood [5, 6, 7]. Such biomarkers may help improve disease stratification, better characterize symptom burden, and potentially support earlier diagnosis or phenotype‐based management.

Among candidate molecules, vascular endothelial growth factor A (VEGF‐A) has attracted considerable attention because of its involvement in angiogenesis, synovial inflammation, cartilage degradation, and osteophyte formation. Previous studies have shown that VEGF‐A levels are elevated in both circulation and synovial fluid in patients with OA, with synovial fluid concentrations generally higher than plasma or serum levels, suggesting a closer relationship to the local joint microenvironment [8]. Moreover, both plasma/serum and synovial fluid VEGF‐A have been reported to correlate positively with Kellgren–Lawrence (KL) grade, whereas synovial fluid VEGF‐A appears to show stronger associations with radiographic severity, joint effusion, and symptom burden [9, 10]. These findings support VEGF‐A as a biologically relevant and clinically promising marker in KOA.

Neuropilin‐1 (NRP1) is another molecule of potential relevance in KOA. NRP1 is a transmembrane co‐receptor that binds several ligands, including semaphorin 3A and VEGF‐A, and enhances VEGF‐A/VEGFR2 signaling [11]. Beyond its established role in vascular biology, emerging evidence suggests that NRP1 may participate in cartilage catabolism, inflammation, and extracellular matrix remodeling in OA [12, 13]. In addition, NRP1 has recently been identified as a facilitator of NGF/TrkA signaling in nociceptors, implicating it in pain sensitization and nociceptive transmission [14, 15]. Since VEGF‐A and NRP1 are biologically linked within signaling networks related to vascular activation, inflammation, and neural sensitization, their combined assessment may provide more comprehensive insight into KOA pathophysiology than either marker alone.

Despite these advances, several important gaps remain. First, most previous KOA biomarker studies have focused on VEGF‐A alone, whereas the clinical relevance of the integrated VEGF‐A–NRP1 axis remains insufficiently defined. Second, the relative performance of circulating and local joint biomarkers has not been fully compared using matched plasma and synovial fluid samples. Third, although KOA pain is increasingly recognized as a multidimensional phenomenon involving both structural pathology and pain sensitization‐related features, few studies have examined whether VEGF‐A and NRP1 are associated with CSI‐defined symptom burden and mechanical pain sensitivity in addition to conventional measures of disease burden. Finally, the potential discriminatory value of exploratory combined biomarker models incorporating VEGF‐A and NRP1 has not been well characterized. Therefore, this study investigated plasma and synovial fluid VEGF‐A and NRP1 levels in patients with KOA, compared their associations with radiographic severity, pain sensitization‐related features, and disease burden, and explored whether individual and combined biomarker models could improve KOA severity stratification.

## 2 Materials and Methods

### 2.1 Ethics Statement

This was a single‐center cross‐sectional observational study. The study was approved by the Ethics Committee of the study hospital and was conducted in accordance with the Declaration of Helsinki. Written informed consent was obtained from all participants prior to enrollment.

### 2.2 Sample Size Calculation

The sample size was estimated based on the primary correlation analyses between VEGF‐A, NRP1, and clinical measures of pain and disease burden. Assuming a moderate correlation coefficient of *r* = 0.30, a two‐sided significance level of 0.05, and 80% statistical power, at least 84 KOA patients were required. To account for potential missing data, sample loss, and assay failure, the final target sample size was increased to 120 patients with KOA.

### 2.3 Participants

A total of 120 patients with KOA who attended the study hospital between August 2023 and August 2025 were enrolled. Patients were eligible if they met all of the following criteria: male or female; age ≥ 45 years; diagnosis of primary KOA according to the American College of Rheumatology criteria [16]; radiographic severity classified as Kellgren–Lawrence (KL) grade 2 to 4 [17]; presence of symptomatic KOA for at least 6 months; ability to complete questionnaires, pain assessment, and sample collection procedures; and provision of written informed consent. In participants with bilateral KOA, the more symptomatic knee was selected as the index knee for clinical assessment and synovial fluid collection. In addition, 53 age‐ and sex‐matched healthy controls who underwent routine health examination at the study hospital were enrolled. Healthy controls had no history of chronic knee pain, no clinical diagnosis of OA or inflammatory arthritis, and normal knee radiographs.

### 2.4 Exclusion Criteria

Participants were excluded if they met any of the following criteria: secondary OA or other forms of arthritis, including rheumatoid arthritis, gouty arthritis, septic arthritis, other inflammatory arthropathies, or crystal‐induced arthritis; previous diagnosis or clinical suspicion of calcium pyrophosphate deposition disease (CPPD) or basic calcium phosphate (BCP) crystal deposition disease; radiographic evidence suggestive of crystal deposition disease, such as chondrocalcinosis, meniscal or articular cartilage calcification, or obvious periarticular calcific deposits; recent knee trauma or previous major surgery involving the index knee; arthroscopic surgery of the index knee within the previous 6 months; intra‐articular glucocorticoid injection within 3 months or hyaluronic acid injection within 6 months before enrollment; systemic glucocorticoid use within the previous 6 months; malignancy, active infection, severe renal insufficiency, or other chronic inflammatory diseases; pregnancy or breastfeeding; severe cognitive impairment; or inability to provide written informed consent or complete study procedures.

### 2.5 Sample Collection

Synovial fluid samples were obtained by aseptic arthrocentesis before any intra‐articular injection or lavage. All synovial fluid samples were processed using the same standardized protocol. Briefly, samples were centrifuged to remove cells and debris, and the resulting supernatants were aliquoted and stored at −80°C until ELISA analysis. Repeated freeze–thaw cycles were avoided. For ethical reasons, synovial fluid was not collected from healthy controls. Peripheral venous blood was collected from the same patients on the same day into EDTA‐containing tubes, centrifuged within 30 min at 1000 × g for 15 min, and the separated plasma was stored at −80°C until analysis.

### 2.6 ELISA

VEGF‐A and NRP1 levels in plasma and synovial fluid were quantified by sandwich ELISA according to the manufacturers' instructions. VEGF‐A was measured using the Nori Human VEGF‐A ELISA Kit (Genorise Scientific, Cat. GR111005; detection range 31–2000 pg/mL; sensitivity 6 pg/mL), and NRP1 was measured using the Human Neuropilin‐1 ELISA Kit (R&D DNRP10; detection range 78.1–5000 pg/mL; sensitivity 9.33 pg/mL). Briefly, standards and samples were added to pre‐coated 96‐well plates, incubated with detection antibody and HRP conjugate, and the absorbance was read at 450 nm. All standards, controls, and plasma and synovial fluid samples were analyzed in duplicate according to the manufacturers' instructions, and the mean value of duplicate wells was used for analysis. According to the manufacturers' data, the intra‐assay and inter‐assay coefficients of variation were 6% and 9% for the VEGF‐A ELISA kit, respectively, and 2.9%–7.3% and 4.9%–6.0% for the NRP1 ELISA kit, respectively. NRP1 concentrations were calculated from the standard curve in pg/mL according to the manufacturer's instructions and then converted to ng/mL for presentation by dividing by 1000.

### 2.7 Clinical Assessment

Pain sensitization‐related features were evaluated using the central sensitization inventory (CSI) and local pressure pain threshold (PPT). The CSI consists of 25 items, each scored from 0 to 4, yielding a total score ranging from 0 to 100, with higher scores indicating a greater CSI‐defined symptom burden [18]. PPT was measured using a digital pressure algometer [19]. Participants were seated in a relaxed position with the index knee flexed at approximately 90°. The anatomical region was standardized to the periarticular area of the index knee. Within this region, the most tender point was identified by palpation, marked before measurement, and used for repeated PPT measurements. A 1‐cm^2^ probe was applied perpendicularly to the skin, and pressure was increased steadily at a rate of approximately 30 kPa/s. Participants were instructed to report the first sensation at which pressure changed to pain, and the corresponding value was recorded as the PPT. Three measurements were obtained at the same marked site with short intervals, and the mean value was used for analysis. Lower PPT values indicate greater mechanical pain sensitivity. Pain intensity was assessed using the visual analog scale (VAS), ranging from 0 (no pain) to 10 (worst imaginable pain), with higher scores indicating greater pain intensity. Disease burden was assessed using the Western Ontario and McMaster Universities Osteoarthritis Index (WOMAC), a 24‐item disease‐specific questionnaire evaluating pain, stiffness, and physical function over the preceding 48 h. The WOMAC total score ranges from 0 to 96, with higher scores indicating greater symptom severity and functional impairment [20].

### 2.8 Statistical Analyses

Statistical analyses were performed using GraphPad Prism 8.0 and Python 3.13. Continuous variables are presented as mean ± standard deviation or median with interquartile range, as appropriate, and categorical variables as number and percentage. Group comparisons were performed using the Kruskal–Wallis test followed by Dunn's multiple‐comparisons test for continuous variables and the chi‐square test for categorical variables. Receiver operating characteristic (ROC) curve analysis was used to evaluate the discriminatory performance of individual biomarkers. Areas under the curve (AUCs), 95% confidence intervals (CIs), optimal cutoff values, sensitivities, and specificities were calculated, with cutoff values determined by maximizing the Youden index. Combined biomarker models were constructed using binary logistic regression after z‐standardization of the included biomarkers. Apparent AUC 95% CIs were estimated using 2000 bootstrap resamples. Internal validation was performed using bootstrap optimism correction with 1000 resamples, and the mean optimism was subtracted from the apparent AUC to obtain the optimism‐corrected AUC. Because these models were developed and internally validated in the same cohort, they were considered exploratory. Spearman's rank correlation analysis was used to assess biomarker–biomarker and biomarker–clinical outcome associations in the overall KOA cohort and within each KL grade subgroup. The Benjamini–Hochberg false discovery rate (FDR) procedure was applied separately to the 64 biomarker–clinical outcome tests and the 16 biomarker–biomarker tests. Both nominal *p* values and FDR‐adjusted *q* values were reported, with *q* < 0.05 considered statistically significant. Multivariable linear regression analyses were performed in patients with KOA. VAS, WOMAC, CSI‐defined symptom burden, and local PPT were entered separately as dependent variables, and each biomarker was evaluated in a separate model adjusted for KL grade, age, sex, BMI, and symptom duration. KL grade was entered as an ordinal covariate. Continuous outcomes and predictors were z‐standardized before analysis; therefore, the reported *β* values represent standardized coefficients. Standardized *β* coefficients, 95% CIs, *p* values, and adjusted *R* ^2^ values were reported. All statistical tests were two‐sided. A nominal *p* value < 0.05 was considered statistically significant, except for the correlation analyses, for which an FDR‐adjusted *q* value < 0.05 was used.

## 3 Results

### 3.1 Baseline Characteristics

Baseline characteristics are summarized in Table 1. No significant differences were observed among healthy controls and patients with KL grade 2, 3, and 4 KOA in age (*p =* 0.124), sex distribution (*p =* 0.396), or BMI (*p =* 0.455), indicating that these baseline characteristics were generally comparable across groups. Among KOA patients, symptom duration differed significantly across KL grade subgroups (*p <* 0.001) and increased progressively with radiographic severity. The distribution of the affected side did not differ significantly among KL grade 2, 3, and 4 patients (*p =* 0.205).

**TABLE 1:** Baseline characteristics of healthy controls and patients with different Kellgren–Lawrence (KL) grades of knee osteoarthritis (KOA).

Variable	Healthy controls (*n* = 53)	KL grade 2 (*n* = 40)	KL grade 3 (*n* = 52)	KL grade 4 (*n* = 28)	*p*
Age, years	63.7 ± 4.0	61.1 ± 8.9	62.9 ± 7.8	64.9 ± 4.9	0.124
Female, *n* (%)	30 (56.6)	28 (70.0)	36 (69.2)	20 (71.4)	0.396
BMI, kg/m^2^	20.8 ± 2.1	21.3 ± 1.9	21.4 ± 2.1	21.5 ± 2.1	0.455
Symptom duration, years	—	3.6 ± 1.8	5.1 ± 2.2	6.4 ± 2.4	< 0.001
Left affected side, *n* (%)	—	28 (70.0)	29 (55.8)	14 (50.0)	0.205

### 3.2 VEGF‐A and NRP1 Levels in Plasma and Synovial Fluid According to KOA Severity

**FIGURE 1:**
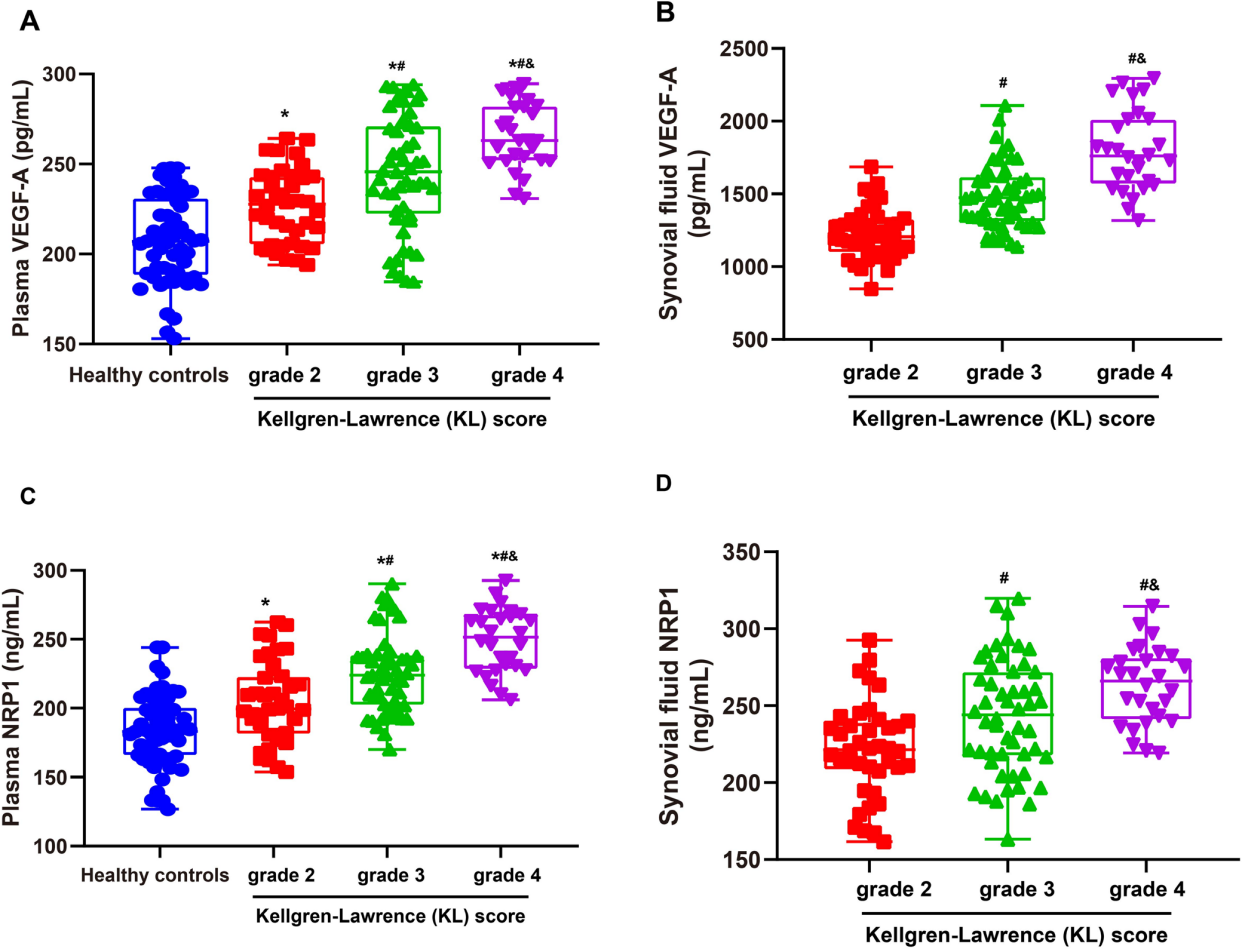
Plasma and synovial fluid levels of VEGF‐A and NRP1 in healthy controls and patients with different Kellgren–Lawrence (KL) grades of knee osteoarthritis (KOA). (A) Plasma VEGF‐A levels in healthy controls and patients with KL grade 2, 3, and 4 OA. (B) Synovial fluid VEGF‐A levels in patients with KL grade 2, 3, and 4 OA. (C) Plasma NRP1 levels in healthy controls and patients with KL grade 2, 3, and 4 OA. (D) Synovial fluid NRP1 levels in patients with KL grade 2, 3, and 4 OA. *p < 0.05 vs. healthy controls; #*p <* 0.05 vs. grade 2; & *p <* 0.05 vs. grade 3.

Plasma VEGF‐A levels differed significantly among healthy controls and patients with KL grade 2, 3, and 4 OA (Figure 1A). Post hoc analysis showed that all OA groups had significantly higher plasma VEGF‐A levels than healthy controls, and significant differences were also observed among the three KL grade groups (all *p <* 0.05). Synovial fluid VEGF‐A levels likewise increased significantly with OA severity (all *p <* 0.001; Figure 1B). Plasma NRP1 levels differed significantly among healthy controls and patients with KL grade 2, 3, and 4 OA (Kruskal–Wallis test, *p <* 0.001; Figure 1C). Post hoc analysis showed that plasma NRP1 levels were significantly higher in all OA groups than in healthy controls and increased progressively with KL grade (all *p <* 0.05). Synovial fluid NRP1 levels also showed a stepwise increase from KL grade 2 to grade 4, with significant differences among all three groups (Kruskal–Wallis test, *p <* 0.001; Figure 1D).

### 3.3 Diagnostic Performance of Plasma and Synovial Fluid VEGF‐A and NRP1 for KOA Severity

**FIGURE 2:**
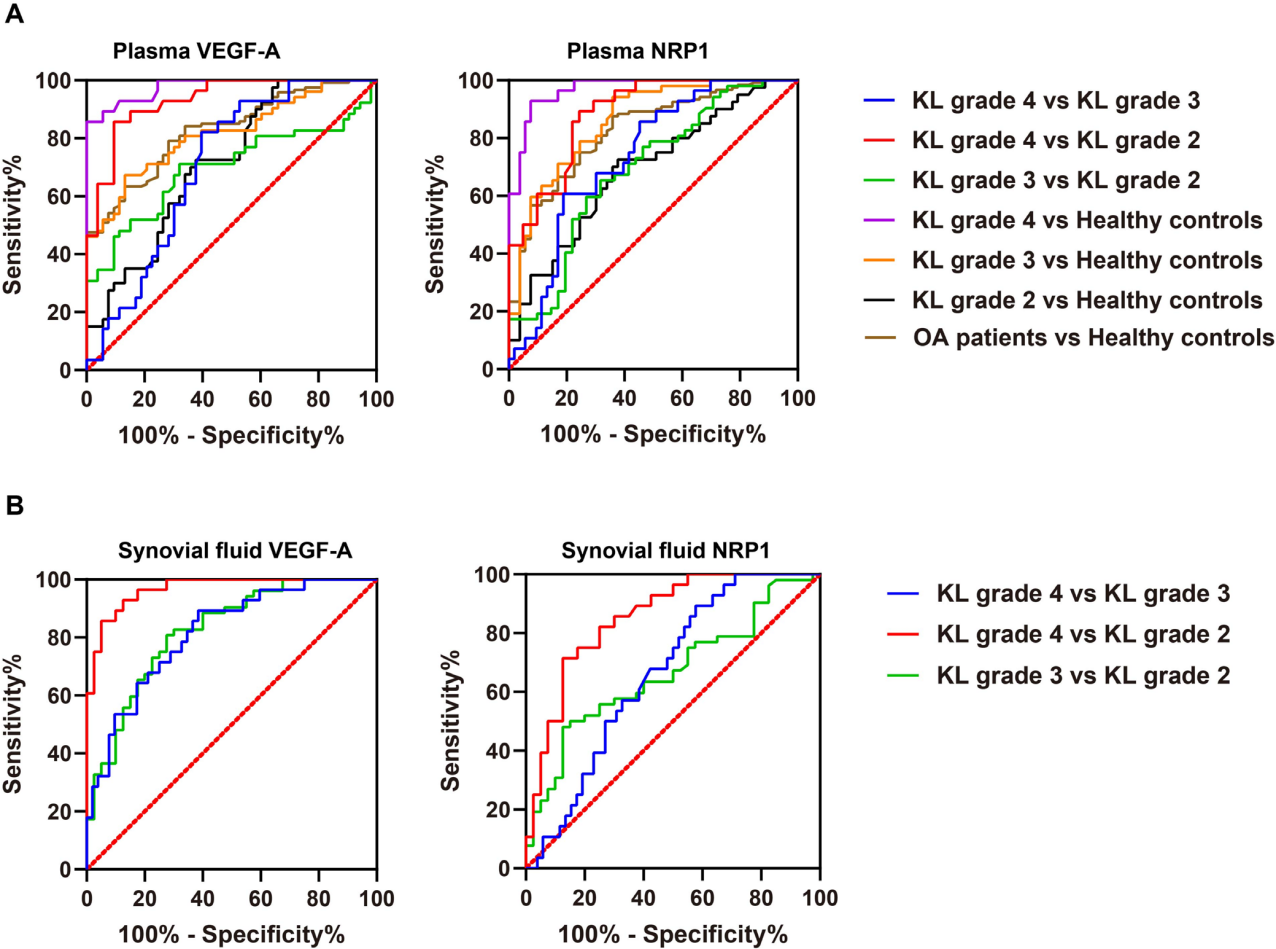
Receiver operating characteristic (ROC) curve analyses of plasma and synovial fluid VEGF‐A and NRP1 in knee osteoarthritis (KOA). (A) ROC curves of plasma VEGF‐A and plasma NRP1 for distinguishing OA patients from healthy controls and for differentiating among Kellgren–Lawrence (KL) grades. (B) ROC curves of synovial fluid VEGF‐A and synovial fluid NRP1 for differentiating OA severity among KL grade 2, 3, and 4 patients.

As shown in Figure 2A and Table 2, ROC analysis demonstrated that plasma VEGF‐A showed good discriminatory performance for distinguishing KOA from healthy controls, with an AUC of 0.819. Its diagnostic performance increased with radiographic severity, with AUCs of 0.712, 0.815, and 0.977 for KL grade 2, 3, and 4 versus healthy controls, respectively. Similarly, plasma NRP1 showed moderate diagnostic ability for distinguishing OA from healthy controls (AUC = 0.749), and its performance also improved with increasing KL grade, reaching an AUC of 0.904 for KL grade 4 versus healthy controls. For synovial fluid biomarkers (Figure 2B; Table 2), VEGF‐A showed good discriminatory ability for OA severity, with AUCs of 0.824 for KL grade 3 versus grade 2, 0.815 for KL grade 4 versus grade 3, and 0.967 for KL grade 4 versus grade 2. Synovial fluid NRP1 showed moderate‐to‐good discrimination across OA severity subgroups, with corresponding AUCs of 0.665, 0.664, and 0.854 for KL grade 3 versus grade 2, KL grade 4 versus grade 3, and KL grade 4 versus grade 2, respectively. Overall, VEGF‐A, particularly in synovial fluid, demonstrated superior discriminatory performance compared with NRP1.

**TABLE 2:** ROC curve analysis of plasma and synovial fluid VEGF‐A and NRP1 for discriminating osteoarthritis and radiographic severity.

Biomarkers	Comparison	AUC	95% CI	Cutoff	Sensitivity (%)	Specificity (%)
VEGF‐A levels						
Plasma (pg/mL)	OA vs. healthy controls	0.819	0.754–0.871	235.4	62.5	86.8
	KL grade 2 vs. healthy controls	0.712	0.608–0.791	213.2	72.5	62.3
	KL grade 3 vs. healthy controls	0.815	0.730–0.878	235.4	67.3	86.8
	KL grade 4 vs. healthy controls	0.977	0.946–0.994	250.5	85.7	100.0
	KL grade 3 vs. grade 2	0.677	0.562–0.785	233.6	71.2	65.0
	KL grade 4 vs. grade 2	0.919	0.850–0.963	250.5	85.7	87.5
	KL grade 4 vs. grade 3	0.699	0.587–0.796	251.7	82.1	59.6
Synovial fluid (pg/mL)	KL grade 3 vs. grade 2	0.824	0.736–0.903	1298.4	80.8	72.5
	KL grade 4 vs. grade 2	0.967	0.925–0.995	1535.8	85.7	95.0
	KL grade 4 vs. grade 3	0.815	0.714–0.902	1512	89.3	61.5
NRP1 levels						
Plasma (ng/mL)	OA vs. healthy controls	0.749	0.669–0.822	199.1	75.0	64.2
	KL grade 2 vs. healthy controls	0.596	0.475–0.712	188.7	72.5	49.1
	KL grade 3 vs. healthy controls	0.784	0.695–0.864	190.7	94.2	50.9
	KL grade 4 vs. healthy controls	0.904	0.832–0.960	216.2	85.7	81.1
	KL grade 3 vs. grade 2	0.690	0.568–0.798	211.3	65.4	67.5
	KL grade 4 vs. grade 2	0.847	0.749–0.926	216.2	85.7	70.0
	KL grade 4 vs. grade 3	0.677	0.557–0.788	246.3	50.0	80.8
Synovial fluid (ng/mL)	KL grade 3 vs. grade 2	0.665	0.554–0.768	247.9	48.1	87.5
	KL grade 4 vs. grade 2	0.854	0.767–0.933	247.8	71.4	87.5
	KL grade 4 vs. grade 3	0.664	0.548–0.773	233.9	89.3	42.3

*Note:* ROC, receiver operating characteristic; Cutoff values were determined using the Youden index.

Abbreviations: AUC, area under the curve; CI, confidence interval; KL, Kellgren–Lawrence; OA, osteoarthritis.

### 3.4 Combined ROC Analyses of VEGF‐A and NRP1 for the Diagnosis and Severity Discrimination of KOA

Combined ROC models were generated using logistic regression and internally validated by bootstrap optimism correction (Table 3). For distinguishing KOA from healthy controls, the plasma VEGF‐A plus plasma NRP1 model showed good performance, with an apparent AUC of 0.853 (95% CI: 0.790–0.904) and an optimism‐corrected AUC of 0.851. For severity stratification, VEGF‐A‐containing models generally performed better than NRP1‐based models. The highest performance was observed for KL grade 4 versus grade 2, where plasma VEGF‐A plus synovial fluid VEGF‐A achieved an apparent AUC of 0.970 (95% CI: 0.928–0.997) and an optimism‐corrected AUC of 0.966, followed by synovial fluid VEGF‐A plus synovial fluid NRP1 with an apparent AUC of 0.964 (95% CI: 0.916–0.992) and an optimism‐corrected AUC of 0.961. For KL grade 3 versus grade 2 and KL grade 4 versus grade 3, VEGF‐A‐containing models showed moderate‐to‐good discriminatory performance, with optimism‐corrected AUCs ranging from 0.810 to 0.818. Mean optimism values were small across models, but these combined models should still be considered exploratory because they were developed and internally validated in a single cohort and require external validation.

**TABLE 3:** Combined ROC models with bootstrap internal validation for the diagnosis and severity discrimination of KOA.

Comparison	Combined model	Apparent AUC	95% CI	Optimism‐corrected AUC	Sensitivity	Specificity
KOA vs. healthy controls	Plasma VEGF‐A + plasma NRP1	0.853	0.790–0.904	0.851	73.30%	88.70%
KL grade 3 vs. 2	Synovial fluid VEGF‐A + synovial fluid NRP1	0.826	0.740–0.910	0.818	82.70%	72.50%
	Plasma VEGF‐A + synovial fluid VEGF‐A	0.824	0.728–0.898	0.815	80.80%	72.50%
	Plasma NRP1 + synovial fluid NRP1	0.704	0.593–0.801	0.692	59.60%	80.00%
KL grade 4 vs. 2	Synovial fluid VEGF‐A + synovial fluid NRP1	0.964	0.916–0.992	0.961	96.40%	85.00%
	Plasma VEGF‐A + synovial fluid VEGF‐A	0.970	0.928–0.997	0.966	100.00%	87.50%
	Plasma NRP1 + synovial fluid NRP1	0.891	0.815–0.959	0.890	67.90%	95.00%
KL grade 4 vs. 3	Synovial fluid VEGF‐A + synovial fluid NRP1	0.819	0.719–0.905	0.812	85.70%	67.30%
	Plasma VEGF‐A + synovial fluid VEGF‐A	0.815	0.711–0.898	0.810	82.10%	69.20%
	Plasma NRP1 + synovial fluid NRP1	0.694	0.562–0.796	0.681	60.70%	75.00%

*Note:* Combined models were generated using logistic regression. Internal validation was performed using bootstrap optimism correction. Apparent AUC, 95% CI, optimism‐corrected AUC, sensitivity, and specificity are shown.

### 3.5 Correlation Analysis of VEGF‐A and NRP1 in Plasma and Synovial Fluid in KOA

**FIGURE 3:**
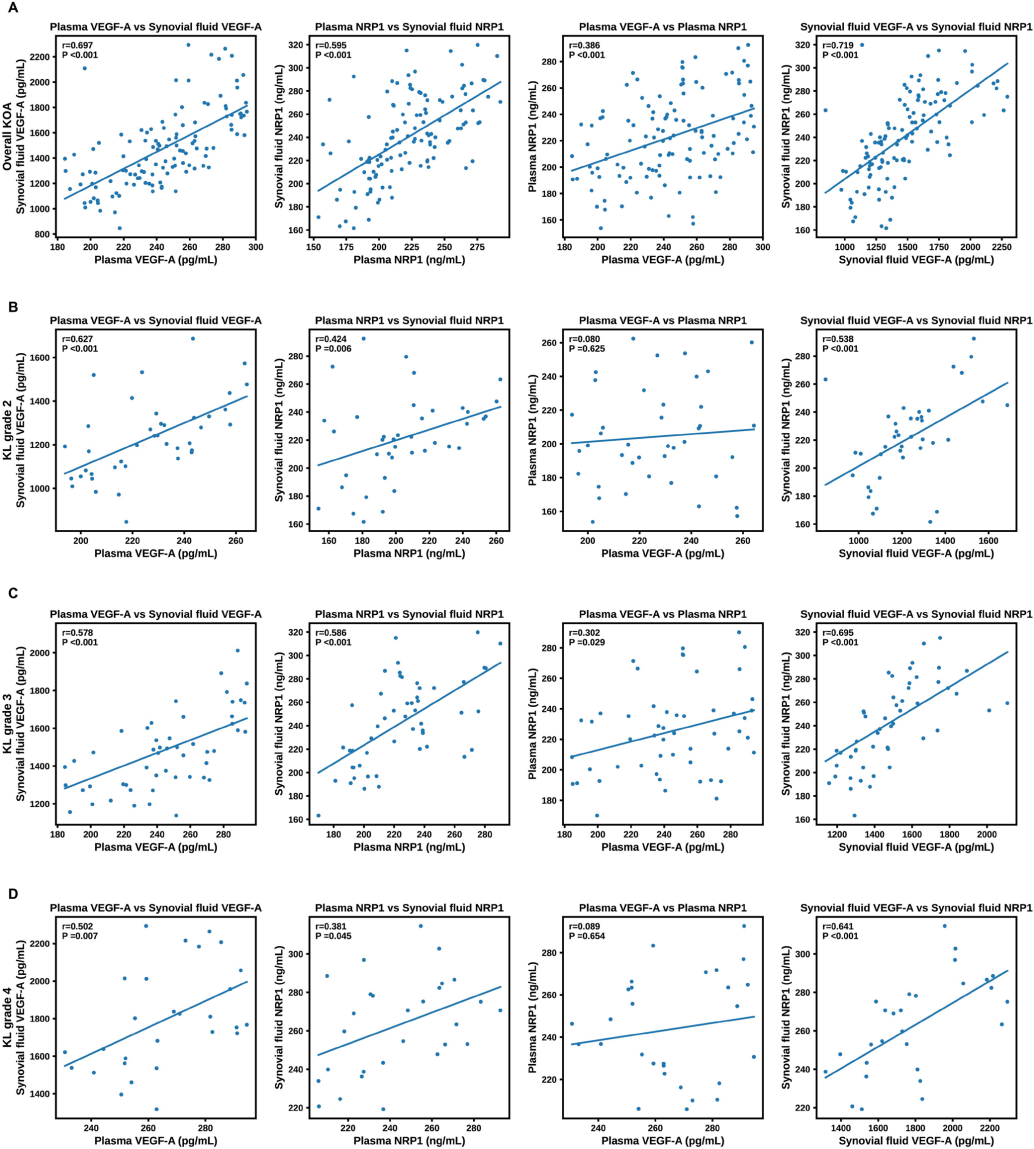
Scatter plots showing the Spearman correlations among plasma VEGF‐A, synovial fluid VEGF‐A, plasma NRP1, and synovial fluid NRP1 in patients with knee osteoarthritis (KOA). (A) Overall KOA cohort (*n* = 120). (B) KL grade 2 subgroup (*n* = 40). (C) KL grade 3 subgroup (*n* = 52). (D) KL grade 4 subgroup (*n* = 28). Spearman correlation coefficients (*r*) and corresponding *p* values are indicated in each plot. *p* values are nominal values.

Spearman correlation analysis showed significant positive associations among plasma and synovial fluid biomarkers in the overall KOA cohort (Figure 3A). After Benjamini–Hochberg FDR correction, plasma VEGF‐A remained significantly correlated with synovial fluid VEGF‐A (*r* = 0.697, *q* < 0.001), plasma NRP1 with synovial fluid NRP1 (*r* = 0.595, *q* < 0.001), plasma VEGF‐A with plasma NRP1 (*r* = 0.386, *q* < 0.001), and synovial fluid VEGF‐A with synovial fluid NRP1 (*r* = 0.719, *q* < 0.001). In KL grade 2 patients, plasma VEGF‐A remained correlated with synovial fluid VEGF‐A (*r* = 0.627, *q* < 0.001), plasma NRP1 with synovial fluid NRP1 (*r* = 0.424, *q* = 0.009), and synovial fluid VEGF‐A with synovial fluid NRP1 (*r* = 0.538, *q* < 0.001), whereas plasma VEGF‐A was not correlated with plasma NRP1 (*r* = 0.080, *q* = 0.654) (Figure 3B). In KL grade 3 patients, all four biomarker pairs remained significant after FDR correction, including plasma VEGF‐A with synovial fluid VEGF‐A (*r* = 0.578, *q* < 0.001), plasma NRP1 with synovial fluid NRP1 (*r* = 0.586, *q* < 0.001), plasma VEGF‐A with plasma NRP1 (*r* = 0.302, *q* = 0.036), and synovial fluid VEGF‐A with synovial fluid NRP1 (*r* = 0.695, *q* < 0.001) (Figure 3C). In KL grade 4 patients, plasma VEGF‐A remained significantly correlated with synovial fluid VEGF‐A (*r* = 0.502, *q* = 0.009), and synovial fluid VEGF‐A remained significantly correlated with synovial fluid NRP1 (*r* = 0.641, *q* < 0.001). By contrast, the correlation between plasma NRP1 and synovial fluid NRP1 did not remain significant after FDR correction (*r* = 0.381, *q* = 0.052), and no significant correlation was observed between plasma VEGF‐A and plasma NRP1 (*r* = 0.089, *q* = 0.654) (Figure 3D). Overall, the consistently stronger association between synovial fluid VEGF‐A and synovial fluid NRP1 suggests a closer relationship between these soluble biomarkers within the synovial fluid compartment.

### 3.6 Associations of VEGF‐A and NRP1 With Pain Sensitization‐Related Features and Disease Burden in KOA

**FIGURE 4:**
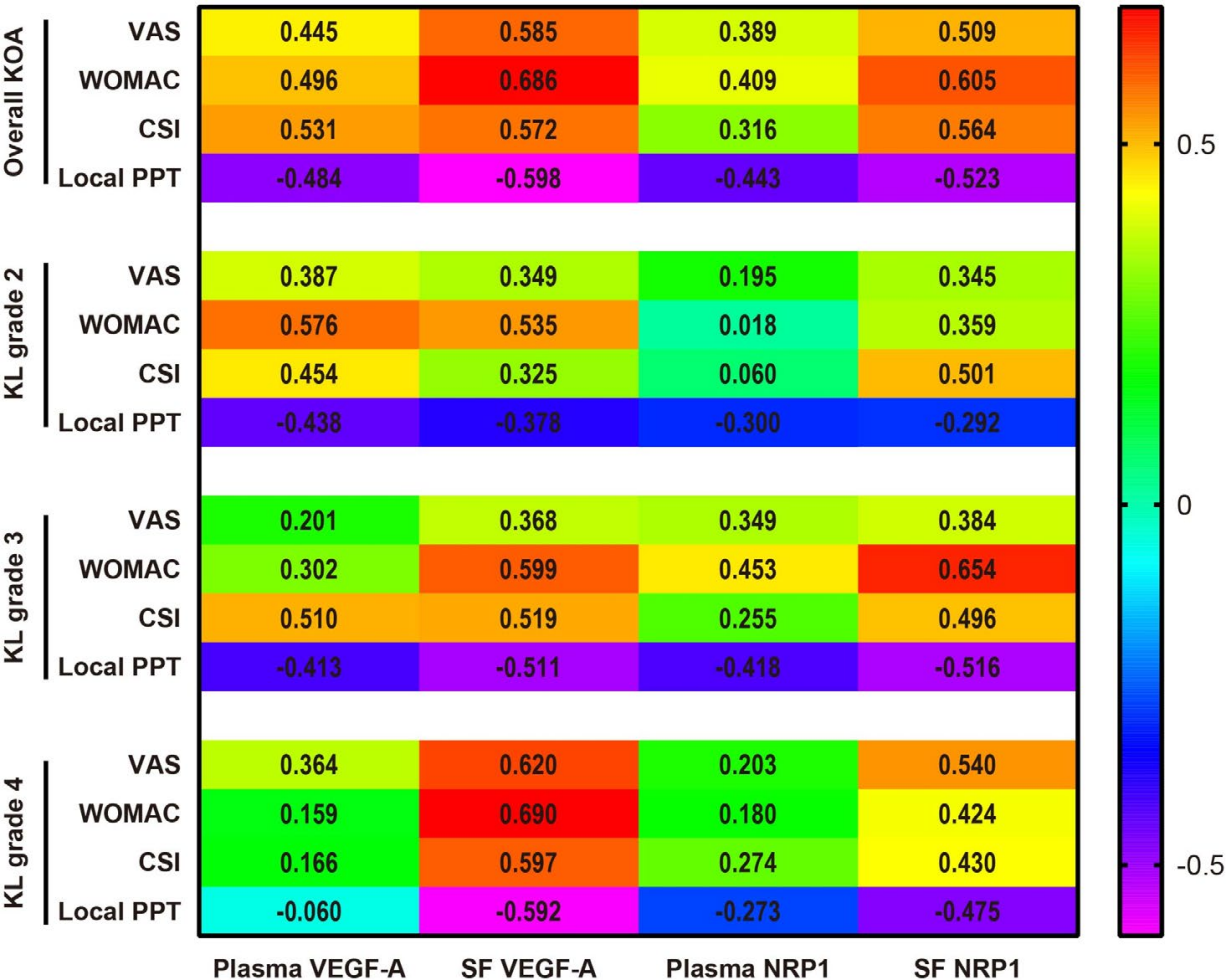
Heatmap of Spearman correlations between plasma and synovial fluid VEGF‐A/NRP1 and clinical outcome measures across knee osteoarthritis (KOA) severity subgroups. Heatmap showing the Spearman correlation coefficients between plasma and synovial fluid VEGF‐A and NRP1 levels and clinical outcome measures in the overall KOA cohort and in each Kellgren–Lawrence (KL) subgroup. The corresponding Spearman *r* values are indicated in each cell.

Spearman correlation analysis showed that both plasma and synovial fluid VEGF‐A and NRP1 were significantly associated with VAS, WOMAC, CSI‐defined symptom burden, and local PPT in the overall KOA cohort (Figure 4 and Table 4). Higher VEGF‐A and NRP1 levels were associated with higher VAS, WOMAC, and CSI‐defined symptom burden, as well as lower local PPT values, indicating greater mechanical pain sensitivity. After Benjamini–Hochberg FDR correction, all biomarker–clinical outcome correlations in the overall KOA cohort remained significant (all *q* ≤ 0.001). Among all biomarker–outcome pairs, synovial fluid VEGF‐A showed the strongest correlations, particularly with WOMAC (*r* = 0.686, *p <* 0.001, *q* < 0.001) and local PPT (*r* = −0.598, *p <* 0.001, *q* < 0.001). In subgroup analyses according to KL grade, the patterns of association differed by disease severity. In KL grade 2, plasma VEGF‐A remained significantly associated with VAS, WOMAC, CSI‐defined symptom burden, and local PPT after FDR correction, whereas plasma NRP1 showed no significant associations. Synovial fluid VEGF‐A remained significantly associated with VAS, WOMAC, and local PPT, while its association with CSI‐defined symptom burden was marginal after FDR correction (*q* = 0.053). Synovial fluid NRP1 remained significantly associated with VAS, WOMAC, and CSI‐defined symptom burden, but not with local PPT after FDR correction. In KL grade 3, synovial fluid VEGF‐A and synovial fluid NRP1 remained significantly associated with all four clinical outcomes after FDR correction. Plasma VEGF‐A remained significantly associated with WOMAC, CSI‐defined symptom burden, and local PPT, but not with VAS; plasma NRP1 remained significantly associated with VAS, WOMAC, and local PPT, but not with CSI‐defined symptom burden. In KL grade 4, correlations for plasma biomarkers were attenuated and were no longer significant after FDR correction. By contrast, synovial fluid VEGF‐A remained significantly associated with VAS (*r* = 0.620, *p <* 0.001, *q* = 0.001), WOMAC (*r* = 0.690, *p <* 0.001, *q* < 0.001), CSI‐defined symptom burden (*r* = 0.597, *p =* 0.001, *q* = 0.002), and local PPT (*r* = −0.592, *p =* 0.001, *q* = 0.002). Synovial fluid NRP1 also remained significantly associated with VAS, WOMAC, CSI‐defined symptom burden, and local PPT after FDR correction. These findings indicate that local joint biomarkers, particularly synovial fluid VEGF‐A, were more consistently associated with pain sensitization‐related features, mechanical pain sensitivity, and disease burden than circulating biomarkers in KOA.

**TABLE 4:** Spearman correlations and FDR‐adjusted significance of plasma and synovial fluid VEGF‐A and NRP1 with pain sensitization‐related features and disease burden indicators according to KOA severity.

Outcome	Plasma VEGF‐A	Synovial fluid VEGF‐A	Plasma NRP1	Synovial fluid NRP1
Overall KOA (*n* = 120)				
VAS	*r* = 0.445; *p <* 0.001; *q* < 0.001	*r* = 0.585; *p <* 0.001; *q* < 0.001	*r* = 0.389; *p <* 0.001; *q* < 0.001	*r* = 0.509; *p <* 0.001; *q* < 0.001
WOMAC	*r* = 0.496; *p <* 0.001; *q* < 0.001	*r* = 0.686; *p <* 0.001; *q* < 0.001	*r* = 0.409; *p <* 0.001; *q* < 0.001	*r* = 0.605; *p <* 0.001; *q* < 0.001
CSI‐defined symptom burden	*r* = 0.531; *p <* 0.001; *q* < 0.001	*r* = 0.572; *p <* 0.001; *q* < 0.001	*r* = 0.316; *p <* 0.001; *q* = 0.001	*r* = 0.564; *p <* 0.001; *q* < 0.001
Local PPT	*r* = −0.484; *p <* 0.001; *q* < 0.001	*r* = −0.598; *p <* 0.001; *q* < 0.001	*r* = −0.443; *p <* 0.001; *q* < 0.001	*r* = −0.523; *p <* 0.001; *q* < 0.001
KL grade 2 (*n* = 40)				
VAS	*r* = 0.387; *p =* 0.014; *q* = 0.021	*r* = 0.349; *p =* 0.027; *q* = 0.038	*r* = 0.195; *p =* 0.229; *q* = 0.257	*r* = 0.345; *p =* 0.029; *q* = 0.039
WOMAC	*r* = 0.576; *p <* 0.001; *q* < 0.001	*r* = 0.535; *p <* 0.001; *q* < 0.001	*r* = 0.018; *p =* 0.912; *q* = 0.912	*r* = 0.359; *p =* 0.023; *q* = 0.033
CSI‐defined symptom burden	*r* = 0.454; *p =* 0.003; *q* = 0.006	*r* = 0.325; *p =* 0.041; *q* = 0.053	*r* = 0.060; *p =* 0.711; *q* = 0.734	*r* = 0.501; *p <* 0.001; *q* = 0.002
Local PPT	*r* = −0.438; *p =* 0.005; *q* = 0.008	*r* = −0.378; *p =* 0.016; *q* = 0.025	*r* = −0.300; *p =* 0.060; *q* = 0.075	*r* = −0.292; *p =* 0.067; *q* = 0.083
KL grade 3 (*n* = 52)				
VAS	*r* = 0.201; *p =* 0.153; *q* = 0.181	*r* = 0.368; *p =* 0.007; *q* = 0.012	*r* = 0.349; *p =* 0.011; *q* = 0.018	*r* = 0.384; *p =* 0.005; *q* = 0.009
WOMAC	*r* = 0.302; *p =* 0.029; *q* = 0.039	*r* = 0.599; *p <* 0.001; *q* < 0.001	*r* = 0.453; *p <* 0.001; *q* = 0.002	*r* = 0.654; *p <* 0.001; *q* < 0.001
CSI‐defined symptom burden	*r* = 0.510; *p <* 0.001; *q* < 0.001	*r* = 0.519; *p <* 0.001; *q* < 0.001	*r* = 0.255; *p =* 0.068; *q* = 0.083	*r* = 0.496; *p <* 0.001; *q* < 0.001
Local PPT	*r* = −0.413; *p =* 0.002; *q* = 0.005	*r* = −0.511; *p <* 0.001; *q* < 0.001	*r* = −0.418; *p =* 0.002; *q* = 0.004	*r* = −0.516; *p <* 0.001; *q* < 0.001
KL grade 4 (*n* = 28)				
VAS	*r* = 0.364; *p =* 0.057; *q* = 0.073	*r* = 0.620; *p <* 0.001; *q* = 0.001	*r* = 0.203; *p =* 0.299; *q* = 0.330	*r* = 0.540; *p =* 0.003; *q* = 0.006
WOMAC	*r* = 0.159; *p =* 0.418; *q* = 0.439	*r* = 0.690; *p <* 0.001; *q* < 0.001	*r* = 0.180; *p =* 0.361; *q* = 0.391	*r* = 0.424; *p =* 0.024; *q* = 0.035
CSI‐defined symptom burden	*r* = 0.166; *p =* 0.400; *q* = 0.426	*r* = 0.597; *p <* 0.001; *q* = 0.002	*r* = 0.274; *p =* 0.158; *q* = 0.183	*r* = 0.430; *p =* 0.022; *q* = 0.033
Local PPT	*r* = −0.060; *p =* 0.760; *q* = 0.772	*r* = −0.592; *p <* 0.001; *q* = 0.002	*r* = −0.273; *p =* 0.161; *q* = 0.183	*r* = −0.475; *p =* 0.011; *q* = 0.018

*Note:* Values are Spearman correlation coefficients (*r*), nominal *p* values, and Benjamini–Hochberg false discovery rate‐adjusted *q* values. *q* < 0.05 was considered FDR significant.

Abbreviations: CSI, central sensitization inventory; KL, Kellgren–Lawrence; KOA, knee osteoarthritis; PPT, pressure pain threshold; VAS, visual analog scale; WOMAC, Western Ontario and McMaster Universities Osteoarthritis Index.

### 3.7 Multivariable Regression Analyses

To determine whether the biomarker–clinical outcome associations were independent of key clinical covariates, multivariable linear regression analyses were performed after adjustment for KL grade, age, sex, BMI, and symptom duration (Table 5). In the adjusted models, plasma VEGF‐A remained significantly associated with VAS, WOMAC, CSI‐defined symptom burden, and local PPT. Plasma NRP1 was independently associated with VAS, WOMAC, and local PPT, but its association with CSI‐defined symptom burden was not statistically significant after adjustment. Synovial fluid VEGF‐A showed the strongest adjusted associations across all outcomes, including VAS (*β* = 0.522, *p* < 0.001), WOMAC (*β* = 0.753, *p* < 0.001), CSI‐defined symptom burden (*β* = 0.676, *p* < 0.001), and local PPT (*β* = −0.622, *p* < 0.001). Synovial fluid NRP1 also remained significantly associated with all four clinical outcomes. These findings suggest that the associations between synovial biomarkers, particularly synovial fluid VEGF‐A, and disease burden or pain sensitization‐related features were not fully explained by radiographic severity, demographic factors, BMI, or symptom duration.

**TABLE 5:** Multivariable linear regression analyses of associations between VEGF‐A/NRP1 and clinical outcomes in patients with KOA.

Outcome	Biomarker	Adjusted standardized *β*	95% CI lower	95% CI upper	*p*	Adjusted *R* ^2^
VAS	Plasma VEGF‐A	0.292	0.114	0.471	0.002	0.282
	Plasma NRP1	0.217	0.036	0.398	0.019	0.252
	Synovial fluid VEGF‐A	0.522	0.317	0.726	< 0.001	0.36
	Synovial fluid NRP1	0.363	0.196	0.530	< 0.001	0.325
WOMAC	Plasma VEGF‐A	0.353	0.175	0.530	< 0.001	0.291
	Plasma NRP1	0.248	0.066	0.430	0.008	0.242
	Synovial fluid VEGF‐A	0.753	0.572	0.935	< 0.001	0.496
	Synovial fluid NRP1	0.449	0.287	0.612	< 0.001	0.362
CSI‐defined symptom burden	Plasma VEGF‐A	0.428	0.257	0.600	< 0.001	0.341
	Plasma NRP1	0.153	−0.033	0.338	0.105	0.216
	Synovial fluid VEGF‐A	0.676	0.485	0.867	< 0.001	0.44
	Synovial fluid NRP1	0.440	0.277	0.603	< 0.001	0.359
Local PPT	Plasma VEGF‐A	−0.313	−0.491	−0.135	< 0.001	0.288
	Plasma NRP1	−0.302	−0.479	−0.125	0.001	0.284
	Synovial fluid VEGF‐A	−0.622	−0.817	−0.427	< 0.001	0.418
	Synovial fluid NRP1	−0.369	−0.536	−0.202	< 0.001	0.326

*Note:* Models were adjusted for KL grade, age, sex, BMI, and symptom duration. *β* values are standardized coefficients.

Abbreviations: CSI, central sensitization inventory; PPT, pressure pain threshold; VAS, visual analog scale; WOMAC, Western Ontario and McMaster Universities Osteoarthritis Index.

## 4 Discussion

In the present study, plasma and synovial fluid VEGF‐A and NRP1 levels were elevated in patients with KOA and increased with radiographic severity. Synovial fluid biomarkers, particularly VEGF‐A, showed stronger associations with radiographic severity, pain sensitization‐related features, and disease burden than circulating biomarkers. Importantly, these associations were further supported by multivariable regression analyses adjusting for KL grade, age, sex, BMI, and symptom duration, suggesting that the biomarker–clinical outcome relationships were not fully explained by radiographic severity or demographic factors. In addition, combined biomarker models improved discrimination of KOA status and radiographic severity, although these models should be regarded as exploratory. Overall, these findings suggest that the VEGF‐A–NRP1 axis, particularly within the synovial fluid compartment, may provide clinically relevant biomarker information for KOA severity assessment and symptom‐related phenotyping.

A major finding of this study is that VEGF‐A increased progressively with KL grade in both plasma and synovial fluid, with synovial fluid VEGF‐A showing superior discriminatory performance and stronger associations with symptom‐related measures. This is broadly consistent with previous studies reporting positive associations between circulating or synovial fluid VEGF‐A levels and radiographic severity in KOA, with synovial fluid VEGF‐A showing closer relationships with local joint pathological changes and symptom burden [10, 21, 22]. The biological relevance of VEGF‐A in KOA is plausible because VEGF‐A is involved in angiogenesis, synovial inflammation, cartilage degradation, and osteophyte formation, all of which are important processes in OA progression [8]. Angiogenesis and neurovascular remodeling have also been implicated in OA‐related pain, providing a possible biological context for the observed associations between synovial fluid VEGF‐A, disease burden, and mechanical pain sensitivity. However, given the cross‐sectional design of this study, these findings should be interpreted as associations rather than evidence of a causal role of VEGF‐A in KOA progression or pain generation.

The findings regarding NRP1 are also noteworthy. Although the overall discriminatory and correlational performance of NRP1 was weaker than that of VEGF‐A, both plasma and synovial fluid NRP1 levels increased with KOA severity, and synovial fluid NRP1 remained significantly associated with pain‐related outcomes after adjustment for clinical covariates. Previous studies have suggested that NRP1 may be involved in inflammatory signaling, cartilage catabolism, extracellular matrix remodeling, and synovial immune activity in OA‐relevant contexts [23, 24]. Earlier work also demonstrated that semaphorin 3A/NRP1 signaling is active in osteoarthritic cartilage [25], while more recent studies suggest that this pathway modulates MMP13 expression, chondrocyte inflammation, apoptosis, and matrix remodeling in OA‐relevant contexts [12, 26, 27, 28]. Nevertheless, the present study measured soluble NRP1 in plasma and synovial fluid rather than membrane‐bound NRP1 expression or signaling activity in joint tissues. Therefore, the mechanistic implications of the NRP1 findings should be interpreted cautiously.

The correlation analyses further suggest a closer relationship between VEGF‐A and soluble NRP1 in synovial fluid than in systemic circulation. In the overall KOA cohort, synovial fluid VEGF‐A showed a stronger correlation with synovial fluid NRP1 than the corresponding correlation observed in plasma. Previous experimental studies have demonstrated that VEGF‐A can bind both VEGFR2 and NRP1, and that NRP1 may enhance VEGF‐A/VEGFR2 signaling involved in endothelial cell migration, survival, and angiogenic sprouting [11]. In inflammatory joint disease, dysregulation of the Sema3A–NRP1–VEGF axis has also been linked to synovial inflammatory activity, supporting the potential relevance of NRP1‐related pathways in joint inflammation [29]. However, because this study measured soluble NRP1 in biofluids, the stronger VEGF‐A–NRP1 correlation in synovial fluid should be interpreted as a closer association between VEGF‐A and soluble NRP1 within the synovial fluid compartment, not as direct evidence of tissue‐level activation of the VEGF/VEGFR2/NRP1 pathway.

Soluble NRP1 may have biological functions distinct from those of membrane‐bound NRP1. It may originate from alternatively spliced NRP1 transcripts and can be secreted as a truncated extracellular isoform capable of binding VEGF165 [30]. Experimental evidence further suggests that soluble NRP1 can act as a decoy receptor by binding VEGF, thereby reducing VEGF binding to endothelial cells and attenuating VEGFR2 activation [31]. Therefore, elevated soluble NRP1 levels in plasma or synovial fluid may reflect altered NRP1‐related pathway activity, inflammation, angiogenesis, or tissue remodeling, but should not be interpreted as direct evidence of membrane‐bound NRP1 signaling activation in joint tissues. Future studies integrating soluble biomarker measurements with synovial tissue immunohistochemistry, cartilage or synovial gene expression analysis, and functional pathway assays are needed to clarify the biological role of NRP1 in KOA.

Another important observation is that synovial fluid biomarkers outperformed plasma biomarkers in most analyses. This pattern may be partly explained by the closer anatomical relationship between synovial fluid and intra‐articular pathological processes, whereas circulating biomarkers may be influenced by systemic dilution and non‐joint sources [10]. Previous OA biomarker studies have similarly reported stronger relationships between synovial fluid markers and disease severity or symptom burden than serum or plasma markers [9, 10]. The present findings extend these observations by showing that synovial fluid biomarkers, particularly VEGF‐A, were also more consistently associated with CSI‐defined symptom burden and local PPT. From a clinical perspective, blood‐based markers may be more feasible for routine use, whereas synovial fluid biomarkers may provide complementary information when joint aspiration is clinically indicated.

The associations with CSI‐defined symptom burden and local PPT are clinically relevant. KOA pain is increasingly recognized as a multidimensional phenomenon that may involve both structural joint damage and altered nociceptive processing. Consistent with this view, previous clinical evidence showed that OA patients with neuropathic pain features had poorer functional capacity and quality of life, further supporting the importance of evaluating symptom‐related phenotypes beyond radiographic severity [2]. Previous KOA research has also emphasized that knee pain, walking performance, chair‐stand ability, and quadriceps function are clinically meaningful outcomes in KOA, further supporting the relevance of symptom and functional burden assessment in this population [32]. In this study, VEGF‐A and NRP1 were associated not only with VAS and WOMAC but also with CSI‐defined symptom burden and local PPT, indicating associations with pain sensitization‐related features and mechanical pain sensitivity. Synovial fluid VEGF‐A showed the strongest associations with WOMAC and local PPT in both correlation and multivariable regression analyses. These findings suggest that higher synovial fluid VEGF‐A levels may be linked to greater disease burden and increased mechanical pain sensitivity. Recent experimental work has also suggested that NRP1 can facilitate NGF/TrkA‐dependent nociceptive signaling, providing biological context for the observed associations between NRP1 levels and pain‐related outcomes in KOA [14, 15]. Nevertheless, because pain sensitization‐related features were assessed using CSI and local PPT rather than a full quantitative sensory testing protocol, these findings should be interpreted as associations with pain sensitization‐related features rather than direct evidence of central sensitization mechanisms.

The application of FDR correction further strengthened the statistical interpretation of the correlation analyses. In the overall KOA cohort, the correlations between plasma or synovial fluid VEGF‐A/NRP1 and VAS, WOMAC, CSI‐defined symptom burden, and local PPT remained significant after Benjamini–Hochberg FDR correction. In subgroup analyses, several associations remained significant after FDR correction, particularly those involving synovial fluid VEGF‐A and synovial fluid NRP1. In KL grade 4 patients, plasma biomarker correlations were attenuated and were no longer significant after FDR correction, whereas synovial fluid VEGF‐A and synovial fluid NRP1 remained significantly associated with pain‐related and disease burden indicators. These findings support the relative robustness of the synovial fluid biomarker associations, while also indicating that subgroup findings, especially those based on smaller samples, should be interpreted cautiously.

The combined ROC analyses suggest that biomarker panels may improve discrimination of KOA status and radiographic severity compared with single markers alone. After bootstrap internal validation, the plasma VEGF‐A plus plasma NRP1 model showed good performance for distinguishing KOA patients from healthy controls. For severity stratification, VEGF‐A‐containing models generally performed better than NRP1‐based models, with plasma VEGF‐A plus synovial fluid VEGF‐A and synovial fluid VEGF‐A plus synovial fluid NRP1 showing the highest performance for distinguishing KL grade 4 from grade 2. The small mean optimism values suggest limited optimism in the apparent AUC estimates. However, because these models were developed and internally validated within a single cohort, they should still be regarded as exploratory and require external validation in independent populations. In addition, although combined plasma and synovial fluid models showed improved discriminatory performance, their routine clinical applicability may be limited because synovial fluid acquisition requires arthrocentesis and is not typically performed solely for biomarker testing. Therefore, plasma‐based models may be more feasible for noninvasive screening or preliminary assessment, whereas synovial fluid‐based or combined plasma–synovial fluid models may provide additional information when joint aspiration is clinically indicated.

Several limitations should be acknowledged. First, this was a cross‐sectional single‐center study, and causality or temporal relationships cannot be inferred. Second, synovial fluid was not collected from healthy controls for ethical reasons, which precluded direct case–control comparisons of synovial biomarkers. Third, although the overall sample size was adequate for the primary correlation analyses, some subgroup analyses may still have been underpowered. Potential confounding factors, such as analgesic use and other clinical variables, were also not fully controlled. In particular, analgesic use, including NSAIDs, acetaminophen, opioids, and topical agents, was not systematically recorded. Because these medications may influence VAS, WOMAC pain scores, CSI‐defined symptom burden, and PPT measurements, residual confounding related to analgesic exposure cannot be excluded. Fourth, pain sensitization‐related features were evaluated using CSI and local PPT, but a comprehensive quantitative sensory testing protocol, including remote PPT, temporal summation, and conditioned pain modulation, was not performed. Therefore, the present findings should be interpreted as associations with pain sensitization‐related features rather than direct evidence of central sensitization mechanisms. Fifth, synovial fluid biomarker concentrations may be influenced by joint effusion volume, dilution effects, and synovial fluid cellularity or leukocyte count. Although all synovial fluid samples were processed using the same standardized protocol, synovial fluid volume, leukocyte count, and total protein concentration were not systematically recorded or used for normalization. Therefore, potential dilution‐ or cellularity‐related effects on synovial VEGF‐A and NRP1 concentrations cannot be excluded. Sixth, occult calcium crystal deposition disease could not be completely excluded. Although patients with a history, clinical suspicion, or radiographic evidence of CPPD or BCP crystal deposition disease were excluded, synovial fluid crystal analysis using compensated polarized light microscopy, Alizarin Red S staining, or electron microscopy was not routinely performed. Therefore, subclinical CPPD or BCP crystal deposition may have remained undetected and could potentially influence synovial biomarker concentrations. Seventh, although all ELISA measurements were performed in duplicate, intra‐ and inter‐assay coefficients of variation were not systematically calculated across all study samples, which should be considered when interpreting the biomarker measurements. Finally, we measured soluble NRP1 in plasma and synovial fluid but did not assess membrane‐bound NRP1 expression or downstream VEGF/VEGFR2/NRP1 or NGF/TrkA signaling activity in synovial or cartilage tissues. Therefore, the present findings cannot directly establish tissue‐level NRP1 pathway activation. The exploratory combined biomarker models were also developed and internally validated in the same cohort and require further external validation.

## 5 Conclusions

In conclusion, plasma and synovial fluid VEGF‐A and NRP1 levels are elevated in KOA and are associated with radiographic severity, pain sensitization‐related features, and disease burden. Synovial fluid VEGF‐A showed the strongest and most consistent associations with clinical outcomes, including after adjustment for KL grade, age, sex, BMI, and symptom duration. Combined biomarker models, particularly those incorporating VEGF‐A, improved KOA diagnosis and severity discrimination, although they should be considered exploratory and require external validation. These findings support the potential value of the VEGF‐A–NRP1 axis as a biomarker framework for KOA severity assessment and symptom‐related phenotyping, while highlighting the complementary role of synovial fluid biomarkers when joint aspiration is clinically indicated.

## Conflicts of Interest

The authors declare no conflicts of interest.

## Data Availability

The data that support the findings of this study are available from the corresponding author upon reasonable request.
